# A Strategy Against Antibiotic‐Resistant Bacteria: Optimizing Prevention of Transmission

**DOI:** 10.1111/apm.70273

**Published:** 2026-11-09

**Authors:** Michael Kemp, Laus Krems Vejrum, Josefine Tange Møller, Asta Lili Laugesen, Marianne Engell Clausen, Anne Line Engsbro

**Affiliations:** ^1^ Clinical Microbiology, Region Zealand Zealand University Hospital—Koege Koege Denmark; ^2^ Department of Clinical Medicine University of Copenhagen Copenhagen Denmark; ^3^ Odense University Hospital Odense Denmark

**Keywords:** antimicrobial resistance, early transmission interruption, infection control, real time surveillance

## Abstract

The rising prevalence of antimicrobial resistance represents a major threat to public health and economic sustainability worldwide. In Europe, vancomycin‐resistant *Enterococcus faecium* (VREfm) and carbapenemase‐producing Enterobacteriaceae (CPE) are of particular concern, as they cause severe infections that are increasingly difficult or impossible to treat. Transmission of VREfm and CPE occurs predominantly in hospital settings, making prevention of nosocomial spread a central component of resistance control efforts. Effective containment relies on rapid detection of transmission events and early intervention to limit onward spread. This requires intensive laboratory surveillance capable of resolving both clonal transmission and horizontal gene transfer, supported by comprehensive sampling of clinical and environmental isolates. In addition, real‐time sharing of isolate‐level data between hospitals is essential to identify and manage cross‐unit and inter‐institutional transmission. Although such surveillance systems require sustained investment, their costs are modest compared with the substantial clinical and economic burden associated with infections caused by antibiotic‐resistant bacteria. In this mini‐review, we outline a strategy for preventing antimicrobial resistance, informed by its implementation in a large Danish healthcare region comprising six public hospitals.

## 1 Introduction

Infections with antibiotic‐resistant bacteria caused 1.27 million deaths in 2019 [1]. Projections predict that the annual number of deaths attributable to antimicrobial resistance may reach almost 2 million people in 2050 [2]. There is a clear global north–south disparity in the risk of serious courses of infections with resistant bacteria [1]. Still, 133,000 people died directly from infections with antibiotic‐resistant bacteria in Europe in 2019 [3].

Despite current efforts to reduce the burden, the number of individuals infected by antibiotic‐resistant bacteria continues to rise, both locally and globally. Current measures are thus clearly insufficient to prevent the increase in antibiotic‐resistant bacteria and new approaches should be considered.

In contrast to currently applied general precautions against spread of pathogens, a new strategy may specifically focus on rapid detection and interruption of transmission of the most important antibiotic‐resistant bacteria.

The strategy should be applied where the bacteria causing the most frequent and serious infections are transmitted. It should include decisions on where to focus intensive monitoring of transmission and which factors have the most significant influence on efficient surveillance. This paper discusses the importance of time to map ongoing transmission, identification of sources, recognition of non‐clonal transmission, and instant sharing of data between hospitals.

In 2022 we described a very small hospital outbreak with *Enterobacter hormaechei* carrying a rare and easily recognizable carbapenemase, OXA‐436 [4, 5]. In the following we use data from that outbreak to illustrate the points made in the text (Table 1).

**TABLE 1:** *bla*OXA‐436 positive enterobacteria in patients hospitalized at the affected ward and hospital environment.

Isolate no.	Sampling date	Specimen	Species	Sequence type	Location of sampling	Department
1	23.08.2017	Swab, wound	*E. hormaechei*	ST90	Odense, G.P.	G.P.
2	18.09.2017	Blood	*E. hormaechei*	ST90	Svendborg Hospital	Internal Medicine
3	10.01.2018	Swab, CVC^a^ insertion site	*E. hormaechei*	ST796	Odense University Hospital	Cardiology
4	16.05.2018	Urine	*E. hormaechei*	ST90	Svendborg Hospital	Urinary Tract Emergency
5	01.12.2018	Urine	*E. hormaechei*	ST90	Nykoebing Falster, G.P.	G.P.
6	21.12.2018	Blood	*E. hormaechei*	ST90	Odense University Hospital	Cardiology
7	29.01.2019	CVC tip	*E. hormaechei*	ST90	Hospital of Southern Jutland	Nephrology
8	21.02.2019	Sink, affected ward	*C. portucalensis*	ST534	Odense University Hospital	Cardiology
9	21.02.2019	Sink, affected ward	*C. portucalensis*	ST534	Odense University Hospital	Cardiology
10	20.03.2019	Blood	*E. hormaechei*	ST90	Odense University Hospital	Intensive Care Department

^a^ Central venous catheter.

Finally, this paper describes how these considerations have been implemented in a large Danish region.

## 2 Strategy Elements

### 2.1 Identify the Most Imminent Threats

Vancomycin‐resistant *Enterococcus faecium* (VREfm) and carbapenemase‐producing Enterobacteriaceae (CPE) are the antibiotic‐resistant bacteria that pose the most serious threat to the population of Northern Europe. VREfm and CPE are listed in the high and critical prioritized group, respectively, of the WHO bacterial priority pathogen list 2024 [6]. They are also included in the most recent CDC list of antimicrobial resistance threats [7].

In Denmark, the proportion of vancomycin‐resistant isolates of invasive *E. faecium* has increased from < 4% to 14% since 2015 [8]. VREfm especially causes infections in patients with severe underlying diseases, including hematological malignancies and conditions requiring prolonged intensive unit treatment. Only a few antibiotics are effective against VREfm and well‐tolerated by the patients. Due to the tendency of VREfm to form biofilm on foreign body material, including intravascular and intravesical catheters and vascular, heart valve, and joint prostheses, cure often necessitates replacement of these materials [9, 10, 11]. VREfm increases length of stay at hospitals compared to vancomycin‐susceptible *E. faecium* [12]. In fact, being colonized with VREfm is itself a risk factor for referral to intensive care and discharge to long‐term hospitalization [13]. Studies have shown that bacteremia with VREfm has a higher mortality rate than bacteremia with vancomycin‐susceptible *E. faecium* , even when adjusted for patient‐related differences [9, 10, 11, 14]. Recent studies have challenged this conclusion [15, 16, 17], and long‐term follow‐up studies are needed to clarify the significance of vancomycin resistance in *E. faecium* bacteraemia on mortality. Parameters other than mortality, summarized above, however, indicate that VREfm places a larger burden on patients and the economy than vancomycin‐susceptible *E. faecium* . The assessment of VREfm as a serious antibiotic‐resistant threat by WHO and CDC emphasizes the need for active efforts against its spread in hospitals.

The number of individuals detected with CPE in Denmark has been increasing steadily for several years [8]. CPE mostly cause urinary tract and abdominal infections, including pyelonephritis, sepsis, and abscesses. In most of the patients in the previously described Danish *E. hormaechei* OXA‐436 outbreak, the first isolate of the CPE from each patient was from sample material indicating severe infections (Table 1).

Often, CPE isolates also carry resistance mechanisms to a number of other antibiotics, making treatment of infections difficult. CPE similar to patient isolates are often found in plumbing installations in hospital wards affected by outbreaks [18, 19, 20, 21, 22]. The genes encoding carbapenemases are often located on mobile genetic elements, especially plasmids, that can transfer resistance across bacterial strains/species (horizontal transfer) [23].

Although VREfm and CPE have some characteristics in common, there are also differences between them: the clinical conversion rate from asymptomatic carrier to clinical infection is higher in CPE than in VREfm [24, 25, 26, 27, 28] and the resulting infection is often more severe. Consequently, the risk of unrecognized active transmission is higher for VREfm than for CPE, which may affect the efficiency of the strategy.

### 2.2 Focus on Place of Transmission

A strategy based on preventing transmission should focus on the location where transmission takes place. VREfm and CPE are almost entirely transmitted in hospitals, where they cause severe complications to treatment of other diseases [8]. Hospital outbreaks with VREfm and CPE occur frequently. Therefore, efforts to limit the spread of VREfm and CPE should focus on hospital transmission.

The OXA‐436 outbreak mentioned above took place in a 1000+ bed public hospital. Until the end of the outbreak, all patients ever identified with CPE producing this carbapenemase in the hospital uptake area had been admitted to the same hospital ward, strongly indicating transmission to take place in that ward only.

### 2.3 Use Extensive Screening for Preventing and Detecting Transmission

Early detection of VREfm and CPE allows rapid intervention, limiting the extent of spread of VREfm and CPE [29]. As VREfm and CPE colonize patients silently in the gut and only occasionally cause clinical disease, active surveillance, including screening of risk patients, is a prerequisite for early detection of transmission [30, 31, 32, 33, 34, 35, 36, 37]. This may be particularly important for detection of VREfm due to the lower carrier to infection rate.

Screening clinical samples for vancomycin‐resistant/vancomycin‐variable *E. faecium* may be organized as a simple and affordable procedure, ensuring identification of manifest or potential VREfm [35]. The procedure, based on PCR detection of resistance genes in primary cultures, is readily adaptable for low‐cost mass screening of CPE.

### 2.4 Establish Surveillance in Close to Real Time

Detection of transmission requires rapid detection of accumulation of closely related bacteria or of identical transferable resistance mechanisms. Thus, surveillance must be based on rapid typing methods with high discriminative power combined with mapping of specific resistance genes and plasmids.

Multi Locus Sequence Typing (MLST) and in particular core genome MLST (cgMLST) based on Whole Genome Sequencing (WGS) has gained status as the gold standard for typing [38, 39, 40, 41]. Several companies and institutions offer excellent software solutions for cgMLST of VREfm and CPE [42, 43, 44]. Typing schemes exist for many bacterial species and new schemes are easily established by users.

Due to a relatively high price and many laboratory steps in preparing DNA for WGS, alternative typing methods may be desirable.

Once outbreak strains have been identified, isolates of bacteria belonging to these strains may easily be recognized by rapid standard methods such as strain‐specific PCR [45, 46, 47].

Fourier Transfer Infrared (FTIR) spectroscopy is an ultra‐rapid and affordable method for comparison of bacterial isolates, which, with recent software improvements, may be applicable for future surveillance of antibiotic‐resistant bacteria [48, 49, 50, 51, 52]. However, more studies are currently needed to characterize the performance of FTIR spectrometry for continuous monitoring.

### 2.5 Include All Available Isolates in Surveillance

Early detection of accumulation of identical bacteria and/or plasmids carrying resistance genes requires characterization of all available relevant bacterial isolates. Failure to include isolates from screening samples or from the environment delays recognition of transmission significantly [34, 53]. Identification of hospital sources of infection requires inclusion of environmental samples in the mapping of transmission; otherwise, risking missing crucial links in outbreaks.

### 2.6 Follow the Genes

Massive amounts of evidence document that horizontal transfer of resistance genes between strains and species is an important factor in dissemination of resistance. Plasmids carrying carbapenemase genes may be detected simultaneously in different strains of enterobacteriaceae [4, 54, 55, 56, 57, 58, 59, 60]. Analysis of plasmid transmission is gradually becoming integrated into surveillance software [61]. Real‐time surveillance of plasmids can reveal complicated multi‐species outbreaks of CPE [62]. At present, plasmid analyses are far from standard procedures, and the results may be difficult to interpret. Routine detection of gene transfer in infection control programs may still be restricted to rare and easily recognizable resistance genes. The *bla*_*OXA‐436*_ gene encoding the OXA‐436 carbapenemase was originally described in three species isolated from four individuals [5], demonstrating this gene's ability to spread widely across bacterial strains. In the *E. hormaechei* OXA‐436 outbreak referred to in Table 1, the third isolate of *E. hormaechei* belonged to a subspecies different from the first two isolates [4]. Interpretation based on chromosomal similarity only would have led to months' delay in the recognition of the outbreak, as the presence of an outbreak was only recognized because of the rare and easily identifiable carbapenemase. Similarly, *Citrobacter portucalensis* isolates from the hospital environment were only related to the outbreak due to an easily recognizable marker, in this case the unusual carbapenemase. The widespread distribution of the *bla*_*OXA‐436*_ gene in this outbreak illustrates the importance of including detailed analyses of extrachromosomal similarities and differences in mapping the spread of resistance.

### 2.7 Share the Data

Specific antibiotic‐resistant bacteria often spread between hospitals. To understand and stop the spread between hospitals, it is essential to compare isolates from multiple hospitals in real time. This requires that local infection control units have immediate access to assembled sequence data and basic genome characterization including typing results, resistome profile, etc. from hospitals other than their own.

Sharing data allows mapping of the epidemiology of VREfm and CPE beyond the local level and identifying genetic characteristics of dominating clones [63]. The first description of the OXA‐436 carbapenemase included strains isolated at six different hospitals [5]. In the previously described OXA‐436 hospital outbreak, the bacteria were isolated from patients who had been transferred to other wards and hospitals or discharged from hospital far from the place of transmission (Table 1). The distance from Odense University Hospital (OUH) to Svendborg Hospital is 45 km. From OUH to Hospital of Southern Jutland (Soenderborg) it is 162 km and from OUH to Nykoebing Falster 172 km.

At the national level, Statens Serum Institut takes care of surveillance of CPE across regions in Denmark. The purpose and approach are different from the strategy described here, and the two surveillance programs should be considered as supplementary to each other.

## 3 Economy

The costs of interventions related to even small hospital outbreaks of antibiotic‐resistant bacteria may be very high [64]. In a recent study, the consequences of real‐time genomic surveillance of healthcare‐associated infections were estimated to result in a 3.2‐fold return of investments over 2 years [65].

VREfm infections increase healthcare costs due to longer hospital stays, need for more complex treatments, expensive antimicrobial therapy, and more intensive nursing care. Several studies have documented the economic burden caused by VREfm [66, 67, 68, 69].

CPE also impose a substantial economic burden through higher healthcare costs, longer hospital stays, increased need for expensive antibiotics, isolation measures, and significant productivity losses from morbidity and mortality, with costs rising proportionally with infection rates. A single CPE infection can cost hospitals a large amount of money, while societal costs caused by CPE may exceed the costs of many chronic diseases [70, 71, 72].

Thus, there appears to be significant savings from implementing strategies preventing the spread of antibiotic‐resistant bacteria in hospitals.

## 4 Implementation in a Large Danish Region

Region Zealand is a Danish region with 850.000 inhabitants. The region runs six public hospitals. All clinical microbiological services, including surveillance of VREfm and CPE, are provided by a single department of clinical microbiology.

During the past 5 years, the considerations discussed above have gradually resulted in the establishment of local rapid WGS of VREfm and CPE. All isolates of VREfm and CPE are now analyzed, regardless of sample material. During the past 5 years, the considerations discussed above have gradually resulted in the establishment of local rapid WGS of VREfm and CPE. All isolates of VREfm and CPE are now analyzed, regardless of sample material. WGS on isolated bacteria colonies is scheduled twice a week. DNA from pure cultures isolated on 5% blood agar plates are extracted using the New England Biolabs Monarch Spin gDNA Extraction kit. A cloudy bacterial solution is achieved by resuspending bacterial colonies in 100 μL PBS. 100 μL Tissue Lysis Buffer and 10 μL Proteinase K are added to CPE samples, and the solution incubates at 37°C and 500 rpm for 1 h. Simultaneously, Enterococci are pre‐treated with 10 μL 50 mg/mL lysozyme at 37°C and 500 rpm for 1 h. Then, 100 μL Tissue Lysis Buffer and 10 μL Proteinase K are added to the solution. Both sample types then undergo a second incubation of 56°C and 1400 rpm for 1,5 h. The remaining protocol is performed according to manufacturer's instructions. DNA libraries for sequencing are constructed using Oxford Nanopore Technologies' rapid barcoding kit (SQK‐RBK114.24). The GridION sequencing runs overnight using latest MinKNOW version and super accurate basecalling. Data are analyzed in minutes by commercial (MBioSEQ Ridom Typer, Ridom, Germany) and in‐house build software pipelines. Results are immediately added to databases available to the regional infection control unit. The region is one administrative unit, allowing simultaneous analyses of isolates across the six hospitals. However, sharing data with other regions is at present prevented by Danish legislation.

The handling of individuals with CPE or VREfm in Region Zealand follows the National Infection Control Guidelines for the prevention of hospital‐acquired infections [73, 74]. Patients with VREfm or CPE are isolated from other patients on the wards in separate rooms. Specific precautions are taken regarding medical equipment, cleaning of the rooms, protective equipment for staff and any visitors, etc. For VREfm, the measures are lifted upon readmission at the earliest after 2 months and negative screening. For CPE, the measures are lifted only after individual assessment in the Infection Control Unit.

Detection of outbreaks, defined as two patients with identical CPO or three patients with the same VREfm, immediately leads to screening of all patients in the ward. When applicable, potential environmental sources are also screened by swabs, and eradication is attempted when positive. Patients from outbreak wards with negative screening results for VREfm are not tested further. Patients from CPE outbreak wards with negative screening are re‐tested upon readmission within 6 months.

## 5 Discussion

The ideas and procedures described in this mini‐review focus on genomic surveillance and rapid detection of transmission as a means to reduce the burden of antibiotic‐resistant bacteria. In hospitals, the success of the strategy depends heavily on active infection control efforts and appropriate interventions, such as isolation of carriers of VREfm and CPE and elimination of environmental sources of infection.

Screening for VREfm and CPE is an essential part of the proposed strategy and includes screening of patients sharing hospital room with patients identified with VREfm or CPE, ward‐level screening during outbreaks and screening of the environment for identification of sources of transmission. Because VREfm is less likely to cause serious illness than CPE, active transmission may easily be overlooked. Application of the strategy to VREfm therefore in particular requires an active screening and isolation policy. Hospital wards with high numbers of at‐risk patients (e.g., hematology departments and intensive care units) and wards with outbreaks of VREfm should be monitored particularly closely with screening and genome sequencing of isolates obtained from screening samples.

## 6 Concluding Remarks

Current surveillance and infection control practices have not prevented an increase in antimicrobial resistance. We here suggest a strategy based on early detection and interruption of transmission, focusing on the most important resistant bacteria and the location where transmission takes place. The strategy implies rapid analyses of isolated resistant bacteria as a routine procedure. Our experience so far shows that implementing the strategy is feasible in a large Danish region.

## Funding

This work was supported by grants from Region Zealand, The Vissing Foundation, and The A.P. Moller Foundation for the Advancement of Medical Science.

## Ethics Statement

The authors have nothing to report.

## Conflicts of Interest

The authors declare no conflicts of interest.

## Data Availability

Data sharing not applicable to this article as no datasets were generated or analyzed during the current study.
